# Electrocardiogram Signal Denoising Using Extreme-Point Symmetric Mode Decomposition and Nonlocal Means

**DOI:** 10.3390/s16101584

**Published:** 2016-09-25

**Authors:** Xiaoying Tian, Yongshuai Li, Huan Zhou, Xiang Li, Lisha Chen, Xuming Zhang

**Affiliations:** School of Life Science and Technology, Huazhong University of Science and Technology, 1037 Luoyu Rd., Wuhan 430074, China; xytianhust@126.com (X.T.); liyongshuai@hust.edu.cn (Y.L.); u201212591@hust.edu.cn (H.Z.); u201212264@hust.edu.cn (X.L.); lishachen00@gmail.com (L.C.)

**Keywords:** electrocardiogram, signal denoising, extreme-point symmetric mode decomposition, nonlocal means

## Abstract

Electrocardiogram (ECG) signals contain a great deal of essential information which can be utilized by physicians for the diagnosis of heart diseases. Unfortunately, ECG signals are inevitably corrupted by noise which will severely affect the accuracy of cardiovascular disease diagnosis. Existing ECG signal denoising methods based on wavelet shrinkage, empirical mode decomposition and nonlocal means (NLM) cannot provide sufficient noise reduction or well-detailed preservation, especially with high noise corruption. To address this problem, we have proposed a hybrid ECG signal denoising scheme by combining extreme-point symmetric mode decomposition (ESMD) with NLM. In the proposed method, the noisy ECG signals will first be decomposed into several intrinsic mode functions (IMFs) and adaptive global mean using ESMD. Then, the first several IMFs will be filtered by the NLM method according to the frequency of IMFs while the QRS complex detected from these IMFs as the dominant feature of the ECG signal and the remaining IMFs will be left unprocessed. The denoised IMFs and unprocessed IMFs are combined to produce the final denoised ECG signals. Experiments on both simulated ECG signals and real ECG signals from the MIT-BIH database demonstrate that the proposed method can suppress noise in ECG signals effectively while preserving the details very well, and it outperforms several state-of-the-art ECG signal denoising methods in terms of signal-to-noise ratio (SNR), root mean squared error (RMSE), percent root mean square difference (PRD) and mean opinion score (MOS) error index.

## 1. Introduction

An electrocardiogram (ECG), derived from the body’s surface, indicates the rhythmic electrical activity of myocardia. The interpretation of abundant physiological and pathological information on the heart contained in ECG recordings provides a noninvasive technique for cardiovascular disease diagnosis. However, ECG signals are usually contaminated with baseline wander, high-frequency noise caused by electromyography, and motion artifacts during collection and transmission. Since these contaminants overlap the cardiac component in both the spatiotemporal and frequency domains, the extraction of weak cardiac components from the corrupted ECG signals and the preservation of the subtle features of the signals are fairly difficult. Indeed, ECG signal denoising is very important for improving the quality of ECG signals, thereby facilitating subsequent signal processing and analysis tasks such as ECG watermarking, compression [1], feature extraction and classification [2,3].

Numerous ECG signal denoising methods have been proposed. These methods can be generally divided into three categories, i.e., spatiotemporal domain methods, frequency domain methods and statistical techniques. Classical low-pass filters [4,5], median filter (MED) [6] and adaptive filtering methods [7,8,9] analyze and reduce the noise in ECG signals in the spatiotemporal domain. These methods generally cannot deliver sufficient noise reduction, especially at high noise corruption. As regards frequency domain methods, wavelet transform-based methods have undergone tremendous development and become a common practice for signal denoising [10,11,12,13,14]. These methods realize noise reduction by shrinking the wavelet transform coefficients of ECG signals and performing inverse wavelet transform of thresholded coefficients. However, in these methods, the base functions are fixed and do not change with the different signals. Meanwhile, the hard thresholding technique may lead to the oscillation of the reconstructed ECG signal while the soft thresholding technique may reduce the amplitudes of the ECG waveforms, especially the R waves [15]. 

Denoising methods based on empirical mode decomposition (EMD) [16], ensemble empirical mode decomposition (EEMD) [17,18] and variational mode decomposition (VMD) [19] have attracted much attention in recent years. The EMD algorithm decomposes the signal into a collection of intrinsic mode functions (IMFs) and a final residue, which is a monotonic first order function. The EMD based denoising method usually produces the denoised result by combining the remaining IMFs except the first several ones with the final residue. This method is difficult to use to adaptively determine the best sifting times and the termination criterion in the gain of every IMF. Furthermore, the final residue will lose evolutionary trend information. These disadvantages will lead to the attenuation of the amplitudes of QRS complexes in denoising ECG signals. As for the EEMD algorithm, it decomposes the complex of ECG signals and the added different white noise by the EMD algorithm and produces the denoised result as the EMD-based denoising method does. Therefore, the problems of the EMD-based denoising method also exist in the EEMD algorithm. For the VMD method, it uses the non-recursive VMD model to determine an ensemble of modes and their respective center frequencies for the input signal such that the combined modes can reproduce this signal. Although the VMD method is more robust to sampling and noise than the EMD method, it will reduce the amplitudes of the ECG waveforms, especially the R waves.

Statistical techniques such as independent component analysis [20], principal component analysis [21] and neural networks [22] have also been applied to ECG noise suppression. These methods are powerful ways to remove in-band noise by discarding the dimensions corresponding to noise, but the models are fairly arbitrary depending on different types of noise and extremely sensitive to the changes in either the signal or the noise.

In 2005, Buades et al. [23] proposed the nonlocal means (NLM) method for image denoising. Based on the assumption that patterns of an image are often self-similar and redundant, the NLM method replaces a pixel considered noisy with the weighted average of all the pixels in the whole image or a pre-defined search window, where the weights are robustly determined by the nonlocal comparison of image patches instead of individual pixels. Because the NLM can achieve adequate noise suppression and excellent detail preservation results, Tracey and Miller have recently applied it to ECG signal denoising [24]. However, the NLM method cannot suppress noise in highly corrupted ECG signals well because it involves inaccurate weight computation based on noisy signals, which will lead to degraded restoration performance. Besides, this method will attenuate the amplitudes of QRS complexes in ECG signals because of the improper weighted averaging. 

To address these issues, we have proposed to combine the extreme-point symmetric mode decomposition (ESMD) [25] with the NLM method for ECG signal denoising. The introduction of ESMD into ECG signal denoising is due to the fact that the ESMD method can automatically determine the termination criterion in the gain of every IMF and the best sifting times, which is the maximum number of IMFs. Because most noise is included in the first several IMFs after the ECG signal decomposition by ESMD, these IMFs will be used for denoising by the NLM method. To ensure good detail preservation, such characteristic waves as the QRS complexes are detected and are preserved during NLM denoising by using relatively small decay parameters. Experimentally, it has been demonstrated that the proposed method has excellent detail preservation performance in the characteristic waves such as the QRS complex, P wave and T wave and good noise reduction performance in the PQ and ST segments of ECG signals. Meanwhile, our method outperforms many existing ECG signal denoising methods by providing a higher signal to noise ratio (SNR) as well as lower root mean squared error (RMSE), percent root mean square difference (PRD) and mean opinion score (MOS) error index. 

## 2. Method

### 2.1. ESMD-Based ECG Signal Decomposition 

The ESMD has been recently proposed by Wang et al. as a modification of the EMD. The ESMD decomposes the signal into a series of intrinsic mode functions (IMFs) and a final residue which is an optimal adaptive global mean (AGM) curve. The IMF is the basic oscillation mode and the AGM curve reflects the evolutionary trend information of the signal. Different from the EMD, the ESMD allows the existence of a certain number of extreme points in the final residue and it determines the best sifting times to obtain the IMFs by optimizing the AGM curve. Using the ESMD method, the ECG signal $S_{n}$ can be decomposed as:

(1) $$S_{n}=\sum_{i=1}^{M}{\mathrm{IMF}}_{i}+R$$

where *M* is the number of IMFs and *R* is the final residue which will be optimized to be an optimal AGM curve.

The detailed implementation of the ESMD based ECG signal decomposition involves the following steps.

Step 1: Find all local extrema points of the ECG signal $S_{n}$, and then connect all the extrema points with line segments. Mark their midpoints and add the boundary points on the left and right, respectively.

Step 2: An interpolating curve based on all the midpoints $\left(m_{1},\ldots ,m_{k})(k\geq 1\right)$ can be constructed. And then the mean value $m^{*}$ of all midpoints can be calculated as:

(2) $$m^{*}=\frac{1}{k}\sum_{i=1}^{k}m_{i}$$

Step 3: Steps 1 and 2 should be repeated with $S_{n}=S_{n}-m^{*}$ until the number of sifting times attains the preset maximum value *T* or $\left|m^{*}\right|\leq 0.001\sigma_{S}$ where $\sigma_{S}$ denotes the standard deviation of ECG signals. Then we can get the first mode IMF_1_.

Step 4: Repeat above steps with $S_{n}=S_{n}-{\mathrm{IMF}}_{1}$ and get the other IMFs until the last residue R has no more than a certain number of extrema points.

Step 5: Repeat the above steps with different *T* in a finite integer interval from *T*_min_ to *T*_max_. Choose *T*_0_ with the smallest variance ratio $v(v=\sigma_{R}/\sigma_{S})$ as the optimal sifting times, where $\sigma_{R}$ denotes the standard deviation of R. 

Step 6: Repeat step 1 to step 4 with the optimal sifting times *T*_0_. In this way, we will obtain all the IMFs and the final residue *R*.

**Figure 1:**
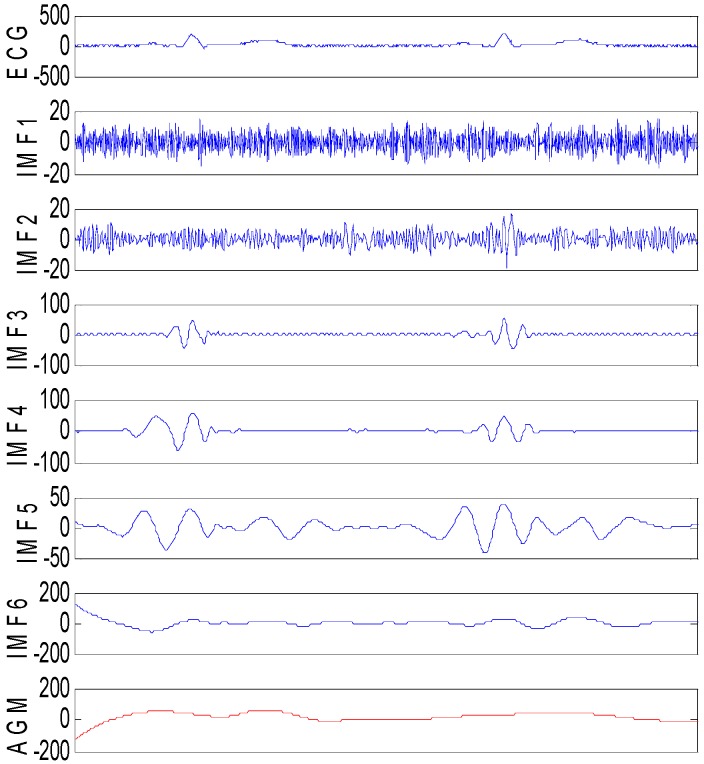
The ESMD method based decomposition of the noisy ECG signal corrupted by Gaussian noise with the variance of 60.

To demonstrate the effect of the ESMD method in decomposing the ECG signal, Figure 1 shows the decomposed results of the simulated ECG signals corrupted by Gaussian noise with the variance of 60. From Figure 1, we can see that the frequency of IMFs decreases with the increasing orders. The high frequency components of ECG signals located in QRS complexes are mainly distributed in the first two IMFs. Here, IMF_1_ contains merely high frequency noise component and there is no apparent correlation between IMF_1_ and the ECG signals. IMF_2_ consists of QRS complex together with high-amplitude noise. IMF_3_~IMF_5_ are slightly stained by high-frequency noise. Other IMFs and AGM contain the low frequency components, and they involve almost no noise. The decomposed results of the ECG signal indicate that it is desirable to only denoise IMF_1_~IMF*_i_* while leave IMF*_i_*_+1_~IMF*_n_* and the residual *R* unprocessed to ensure effective noise reduction and good detail preservation.

### 2.2. QRS Detection 

When the ECG signals are contaminated by high levels of noise, the noise will be decomposed into IMF_1_ and IMF_2_ as shown in Figure 1. Because noise will submerge the QRS complex components in IMF_1_ and IMF_2_, it is preferable to extract the exiguous QRS complex components while suppress the noise. However, this is a very difficult task. If the NLM filter is directly implemented on them, the noise and the ECG components will be removed simultaneously. Therefore, we propose to detect the location of QRS complexes by the wavelet based detection algorithm. Using this algorithm, we will obtain the set *P* of points in the QRS complexes.

As regards the detection of R wave, the wavelet transform is applied to IMF_1_ and IMF_2_, and the singularity is found in the wavelet domain. Then the mean amplitudes of the poles are obtained by setting the threshold of R wave to be 30% of the difference value between the maximum amplitudes and the minimum ones. Finally, the distance between two adjacent maxima is computed. If this distance is less than 0.4, the maxima with lower amplitude will be removed. In this way, the points in the R wave will be obtained.

For the detection of the Q wave, if the wave is a forward/inverted wave, a minimum/maximum forward from the R wave is found in the wavelet transform domain. The detection of the S wave is similar to that of the Q wave. The only difference is that the S wave is found backward from the R wave in the wavelet transform domain.

### 2.3. NLM Denoising of IMFs 

**Figure 2:**
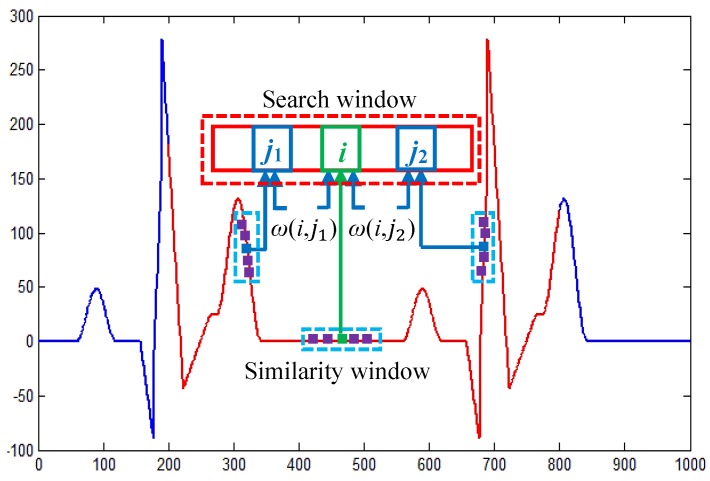
The principle of the NLM method.

For ECG signal denoising, the NLM method restores a given point using the weighted average of point intensities within a search window Ω, where the weights are determined based on the similarity between two windows centered at the two considered points. The principle of the NLM method is shown in Figure 2.

Let the ECG signal $S_{n}$ be $S_{n}=S_{o}+\eta$, where $S_{o}$ and η denotes the noise-free ECG signal and noise, respectively. Mathematically, the restored intensity $S_{NLM}(i)$ of the *i*th point by the NLM method is computed as:

(3) $$S_{NLM}(i)=NL(S_{n})(i)=\frac{1}{C(i)}\sum_{q\in \Omega }\omega (i,q)\cdot S_{n}(q)$$

where $C(i)=\sum_{q\in \Omega }\omega (i,q)$ is the normalization constant, and $S_{n}(q)$ is the intensity of the point centered at q in the noisy ECG signal; ω(i,q) is the weight measured by the difference of the similarity windows N(i) and N(q) centered at i and q, respectively. The weight ω(i,q) is computed as:

(4) $$\omega (i,q)=\exp(-\frac{{\left\|S_{n}(N(i))-S_{n}(N(q))\right\|}_{2,a}^{2}}{h^{2}(i)})$$

where ${\left\|S_{n}(N(i))-S_{n}(N(q))\right\|}_{2,a}^{2}$ means the weighted distance between $S_{n}(N(i))$ and $S_{n}(N(q))$ with a denoting the standard deviation of the Gaussian kernel, and h(i) denotes the decay parameter.

The decay parameter has an important influence on the restoration performance of the NLM method. To expound the choice of this decay parameter for the effective ECG signal denoising, we will introduce the bias-variance principle [26]. Based on this principle, the corresponding mean square error (MSE) for the denoised result $S_{NLM}(i)$ can be expressed as the sum of the squared bias term $bias^{2}(S_{NLM}(i))$ and the variance $var(S_{NLM}(i))$:

(5) $$\mathrm{MSE}(S_{NLM}(i))=bias^{2}(S_{NLM}(i))+var(S_{NLM}(i))$$

The two items $bias^{2}(S_{NLM}(i))$ and $var(S_{NLM}(i))$ are both relevant to h(i). According to [26], $bias^{2}(S_{NLM}(i))$increases and $var(S_{NLM}(i))$ decreases with the increasing h(i), because smaller $bias^{2}(S_{NLM}(i))$ means better noise suppression performance while smaller $var(S_{NLM}(i))$ means better detail preservation performance. Therefore, h(i) should change with different ECG signal points to obtain good restoration performance. For the points in the PQ and ST segments of ECG signals, h(i) should be large to ensure sufficient noise reduction by producing relatively small $bias^{2}(S_{NLM}(i))$. On the contrary, h(i) should be small for the points in the characteristic waves such as the P wave, QRS complex and T wave to provide good sharpness preservation by producing relatively small $var(S_{NLM}(i))$. Based on the above analysis, this parameter $h_{j}(i)$ will be determined for different IMFs according to the QRS detection results, i.e.,

(6) $$h_{j}(i)=\left\{ \begin{array}{ll} h_{QRS,j} & i\in P\\ h_{j}(i) & i\notin P \end{array}\right.$$

where $h_{QRS,j}$ denotes the decay parameter for the points in the QRS complex of IMF*_j_*. Inspired by the work in [27,28], the parameters $h_{j}^{2}(i)$ and $h_{QRS,j}^{2}$ will be chosen to be proportional to the estimated noise variance $\delta^{2}$. Because the noise will become weaker and weaker with the increasing orders of IMFs, the parameter $h_{j}^{2}(i)$ will be chosen adaptively for each IMF*_j_* as $h_{j}^{2}(i)=(c\cdot \delta^{2})/j$ where c is a constant with 0.5<c<1.5. Considering that $h_{QRS,j}$ should be smaller than $h_{j}(i)$ to ensure good signal sharpness preservation, we will define it as $h_{QRS,j}^{2}=(c\cdot \delta^{2})/(3j)$. As regards δ, it is estimated using the median absolute deviation (MAD) method as:

(7) δ=1.4826×MAD(R)=1.4826×median(|D−median(D)|)

where median denotes the median operator, and D={D(1),⋯,D(l),⋯D(L)} is the set of the local residuals of the selected homogenous region of length *L* in the noisy signal $S_{n}$ with $D(l)=(2S_{n}(l)-(S_{n}(l-1)+S_{n}(l+1)))/\sqrt{6}$. 

## 3. Implementation of Our Method

**Figure 3:**
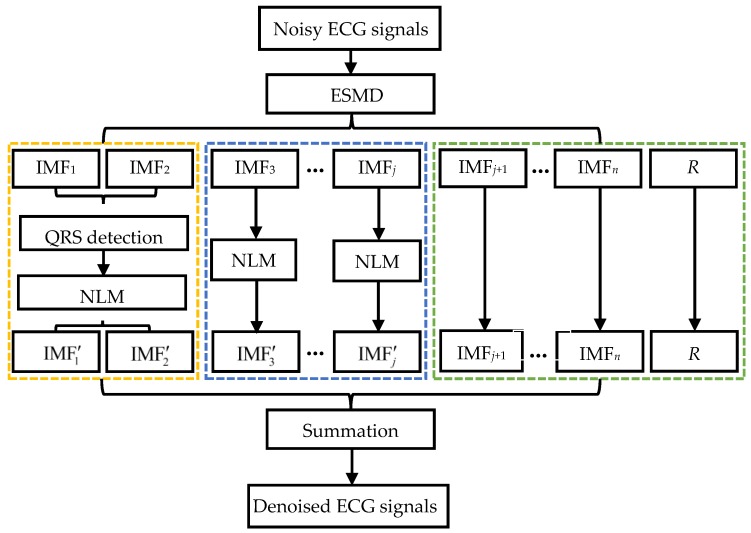
The flowchart of the ESMD-based NLM method for ECG signal denoising.

The implementation of the ESMD-based NLM denoising method is given in Figure 3. It involves the following three steps.

Step I. The given ECG signals are decomposed into several IMFs and AGM using the ESMD method.

Step II. The different IMFs with different orders are processed as follows. 

II-1. The QRS complexes in IMF1 and IMF2 are detected and the decay parameter is adaptively determined for each point in IMF1 and IMF2 based on the QRS detection result. IMF1 and IMF2 are filtered by the NLM method using the point-wise decay parameter. 

II-2. IMF_3_ ~ IMF*_j_* are filtered by the NLM method using the adaptive decay parameter, where *j* will be chosen flexibly according to the levels of noise. 

II-3. IMF*_j_*_+1_ ~ IMF*_n_* and AGM are kept intact.

Step III. The denoised results ${\mathrm{IM}F\prime }_{1}$~${\mathrm{IM}F\prime }_{j}$ of ${\mathrm{IMF}}_{1}$~${\mathrm{IMF}}_{j}$, unprocessed IMF*_j_*_+1_~IMF*_n_* and AGM are combined to reconstruct the final denoised ECG signals.

## 4. Experimental Results

To evaluate the performance of the proposed ESMD-based NLM (ESMD-NLM) method, we have chosen four other denoising methods for comparison including the NLM method [24], the VMD method [29], the EEMD method [17] and the median (MED) filter [6]. Experiments have been conducted on the simulated ECG signals corrupted with Gaussian white noise and muscle artifact (MA) noise as well as the real ECG signals downloaded from MIT-BIH database, QT database and MIH-BIH ECG compression test database [29]. The performance of the five methods is evaluated by such metrics as SNR, RMSE, PRD and MOS_error_ index [30]. The higher SNR value and lower RMSE, PRD and MOS_error_ values mean better signal restoration performance. Here, the MOS_error_ index is used to measure signal distortion based on the clinical blind and semiblind MOS tests [30]. The blind and semiblind tests are performed to obtain the cardiologists’ evaluation of the denoised signals from different denoising algorithms without knowing the source of each tested signal and without knowing the denoising method, respectively. The MOS_error_ value is computed as the mean of the results of the blind and semiblind tests of three independent cardiologists for the simulated ECG signals [30]. The other metrics are defined as:

(8) $$\mathrm{SNR}=10\cdot \mathrm{lo}g_{10}\left[\frac{\sum_{i=1}^{m}{\left(S_{o}(i)\right)}^{2}}{\sum_{i=1}^{m}{\left(S_{NLM}(i)-S_{o}(i)\right)}^{2}}\right]$$

(9) $$\mathrm{RMSE}=\sqrt{\frac{\sum_{i=1}^{m}{\left(S_{NLM}(i)-S_{o}(i)\right)}^{2}}{m}}$$

(10) $$\mathrm{PRD}=\sqrt{\frac{\sum_{i=1}^{m}{\left(S_{NLM}(i)-S_{o}(i)\right)}^{2}}{\sum_{i=1}^{m}{\left(S_{o}(i)\right)}^{2}}}\times 100$$

where *m* is the length of the ECG signals. 

### 4.1. Parameter Setting

As regards the NLM method, the decay parameter is fixed at 1.1δ. The half-width of the search window and that of the similarity window in the NLM and ESMD-NLM methods are chosen to be 500 samples and 10 samples, respectively. For the VMD method, the moderate bandwidth constraint is 2000, the noise-tolerance is 0, the number of modes is 7~10 and the initialized center frequencies of all modes are 8~11. As for the EEMD method, the standard deviation of the added white noise is 0.02, the number of realizations is 500 and the maximum number of sifting iterations is 5000. For the median filter, the filtering window is fixed at 5 samples. 

### 4.2. Comparison of Restoration Performance

#### 4.2.1. Comparison Based on the Simulated ECG Signals 

For the simulated ECG signals, Gaussian white noise with the variance $\sigma^{2}$ of 20, 30, 40, 50 and 60, and muscle artifact noise with the variance of 60 will be added to the noise-free ECG signals. Here, the simulation of the uncorrupted ECG signals and two kinds of noise is done using the Open-Source Electrophysiological Toolbox (OSET). This toolbox implemented in MATLAB is available at http://oset.ir/category.php?dir=Tools. 

**Figure 4:**
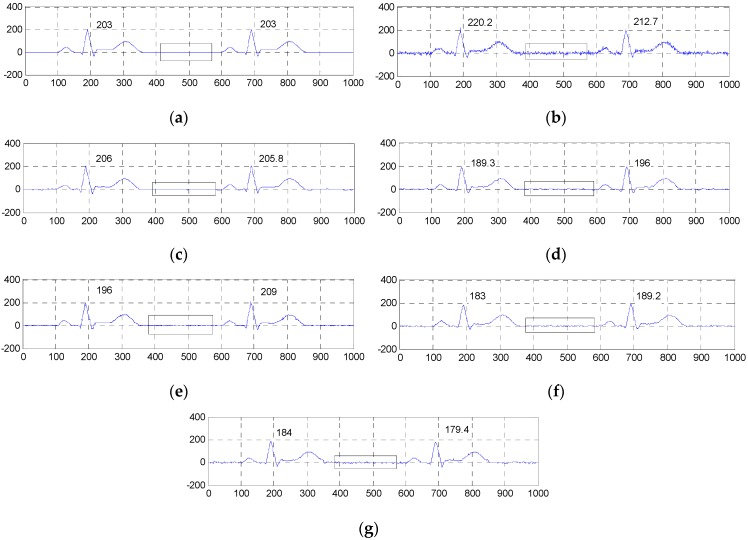
Comparison of denoised results of the ESMD-NLM method and other four methods operating on the simulated ECG signal. (**a**) The original ECG signal, (**b**) the noisy ECG signal (Gaussian white noise, $\sigma^{2}$ = 50), (**c**) the ESMD-NLM method, (**d**) the EEMD method, (**e**) the NLM method, (**f**) the VMD method, (**g**) the MED filter. The digits and the black box represent the values of amplitudes and the P-Q and S-T segments of ECG signals, respectively.

**Figure 5:**
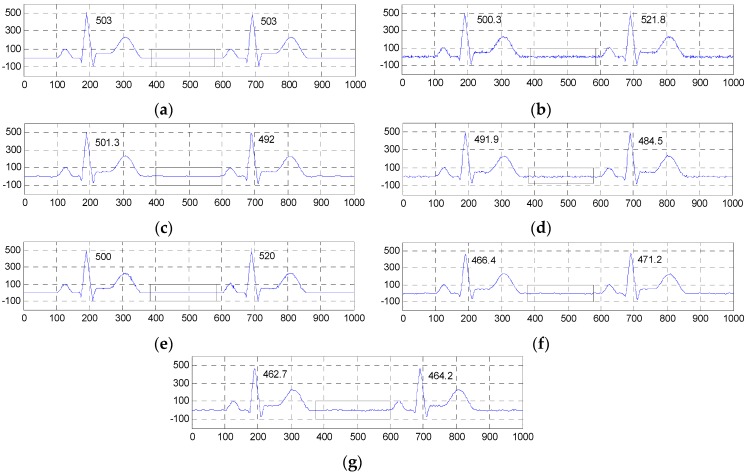
Comparison of denoised results of all evaluated methods operating on another simulated ECG signal. (**a**)Tthe original ECG signal, (**b**) the noisy ECG signal (Gaussian white noise, $\sigma^{2}$ = 50), (**c**) the ESMD-NLM method, (**d**) the EEMD method, (**e**) the NLM method, (**f**) the VMD method, (**g**) the MED filter.

Figure 4 and Figure 5 show the two groups of simulated ECG signals, the ECG signals corrupted by Gaussian white noise with a variance of 50 and the denoised results for the five methods. 

To demonstrate the advantage of our method, we will focus the performance evaluation on the restored results of smooth segments and the ECG’s amplitudes. From Figure 4 and Figure 5, we can see that the smooth segments remain very smooth when processed by the ESMD-NLM method, but some noise still remains in these segments for the MED, EEMD, VMD and NLM methods. As for the amplitudes, the values of the first amplitudes in the first simulated ECG signal and the denoised results are 203, 206, 189.3, 196, 183 and 184, and the differences of amplitudes between the corresponding denoised signals and the original signal are 3, 13.7, 7, 20 and 19 for the ESMD-NLM, EEMD, NLM, VMD and MED methods, respectively. Apparently, our method outperforms the compared methods in terms of the preservation of ECG’s amplitudes. The above comparison indicates that the proposed method has excellent detail preservation performance in the characteristic waves such as the QRS complex, P wave and T wave and good noise reduction performance in the PQ and ST segments of ECG signals. The reason will be explained below. Our method can preserve QRS complexes very well due to the introduction of the QRS detection algorithm and the adoption of a small decay parameter. Meanwhile, the proposed method can deliver sufficient noise reduction because of the utilization of a relatively large decay parameter for the smooth segments. 

**Figure 6:**
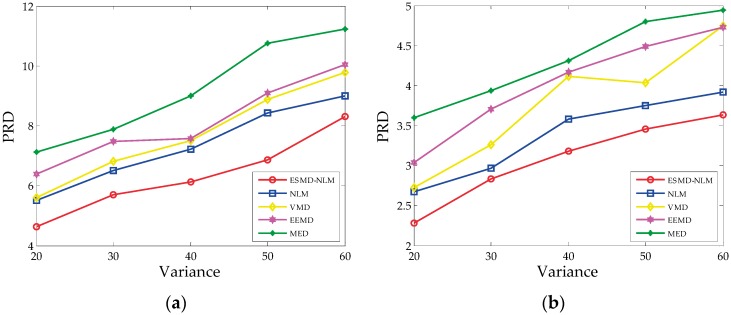
The PRD for the two groups of simulated ECG signals filtered by all evaluated methods when the variance $\sigma^{2}$ of Gaussian noise is 20, 30, 40, 50 and 60, respectively. (**a**) The first group; (**b**) the second group.

**Figure 7:**
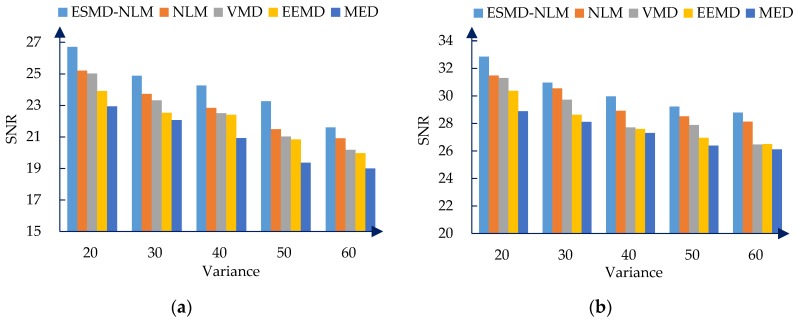
The SNR for the two groups of simulated ECG signals filtered by all evaluated methods when the variance $\sigma^{2}$of Gaussian noise is 20, 30, 40, 50 and 60, respectively. (**a**) The first group. (**b**) The second group.

**Figure 8:**
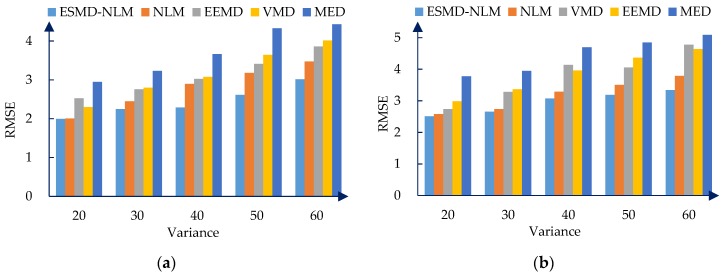
The RMSE for the two groups of simulated ECG signals filtered by all evaluated methods when the variance$\sigma^{2}$of Gaussian noise is 20, 30, 40, 50 and 60, respectively. (**a**) The first group. (**b**) The second group.

Figure 6, Figure 7 and Figure 8 show the PRD, SNR and RMSE comparisons for all the evaluated methods operating on the simulated ECG signals corrupted with Gaussian white noise of different variances. Table 1 shows the mean MOS_error_ values for all evaluated methods. The mean MOS_error_ is computed as the mean of all MOS_error_ values for each compared denoising method when $\sigma^{2}$ = 20, 30, 40, 50 and 60. It can be seen that from Figure 6, Figure 7 and Figure 8 and Table 1 that for the various noise variances, the ESMD-NLM method outperforms all other compared methods by providing much higher SNR values and much lower PRD, RMSE and MOS_error_ values. Here, it should be noted that the MOS_error_ value of the MED method reaches 30 while the MOS_error_ values of the ESMD-NLM method and the VMD and EEMD methods are lower than 15 and 30, respectively. Based on the classification criterion of the signal quality in [30], it can be seen that the quality of the denoised signals for the MED method is bad. The quality of the denoised signals for the ESMD-NLM method is very good and it is better than that for the VMD and EEMD methods, although the latter is good to some extent. The above comparison indicates that the ESMD-NLM method can provide the highest-quality denoised signals by suppressing Gaussian white noise and preserving the details much better than the other compared methods. The superiority of the proposed method results from the fact that it separates the noise-free signals from noise by decomposing the signals into several IMFs using the ESMD, which can attenuate the influence of noise and thus facilitate determining the weight in the NLM method accurately.

**Figure 9:**
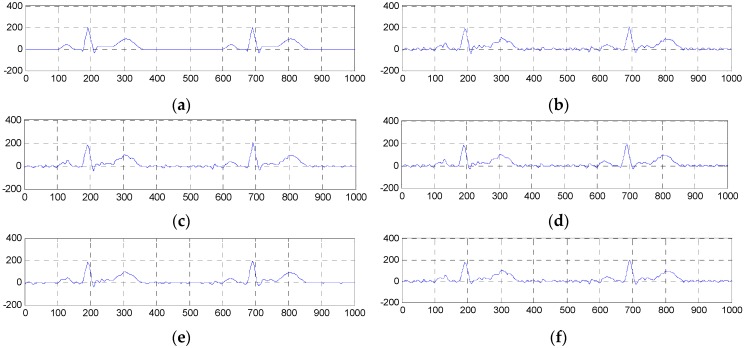
Comparison of denoised results of the ESMD-NLM method, the NLM method, the EEMD method and the VMD method operating on the simulated ECG signal corrupted by muscle artifact noise with the variance of 60. (**a**) The original ECG signal, (**b**) the noisy ECG signal (muscle artifact noise, $\sigma^{2}$ = 60), (**c**) the ESMD-NLM method, (**d**) the EEMD method, (**e**) the NLM method, (**f**) the VMD method.

To further demonstrate the effectiveness and advantage of the proposed method in removing other kinds of noise, we have conducted experiments on the first group of simulated ECG signals corrupted by muscle artifact noise with the variance of 60. Here we will only make comparisons among the ESMD-NLM method, the VMD method, the EEMD method and the NLM method because the latter are closely related to our method. Figure 9 shows the simulated ECG signals, the noisy ECG signals and the denoised results for the above four methods. From Figure 9, we can see that the smooth segments in the noisy ECG signals keep more smooth when processed by the ESMD-NLM method than by other methods. Meanwhile, the ESMD-NLM method can generally maintain the shape of the QRS complex, the P wave and the T wave better than the compared methods. The above visual comparison indicates the advantage of the proposed method over the VMD, EEMD and NLM methods in denoising the ECG signals corrupted with muscle artifact noise.

Table 2 shows the PRD, SNR, RMSE and MOS_error_ comparisons for the ESMD-NLM method and the other three compared methods operating on the first group of simulated ECG signals corrupted with MA noise. As shown in Table 2, the SNR values of the EEMD, VMD and NLM methods are lower than those of the ESMD-NLM method, and their PRD, RMSE and MOS_error_ values are higher than those of the proposed method. Please note that the MOS_error_ values of the VMD and EEMD methods are higher than 50, which indicates the very bad quality of the denoised signals for the two methods. Likewise, the NLM method provides the bad quality denoised signals because its MOS_error_ value reaches 30. By comparison, the proposed method can provide the denoised signals with good quality. Indeed, the data in this table illustrate that the ESMD-NLM method can suppress muscle artifact noise and preserve the details better than other methods. 

#### 4.2.2. Comparison Based on the Real ECG Signals 

To demonstrate the practicality of the proposed method, we have applied it to denoising the three groups of real ECG signals. Here, we have selected the 100m.dat, 111m.dat and 221m.dat from MIT-BIH Arrhythmia Database as the first group, the sel100m.dat, sel117m.dat and sel114m.dat from QT database as the second group, and the 13420_12m.dat, 13649_04m.dat and 12713_04m.dat from MIH-BIH ECG Compression Test Database as the third group. For the real ECG signals in units of mV, the gain is 200 and 400 for the first and second groups and the third group, respectively. The base is 1024 and the sampling frequency is 360 Hz for the first group while the base is 0 and the sampling frequency is 250 Hz for the second and third groups.

**Figure 10:**
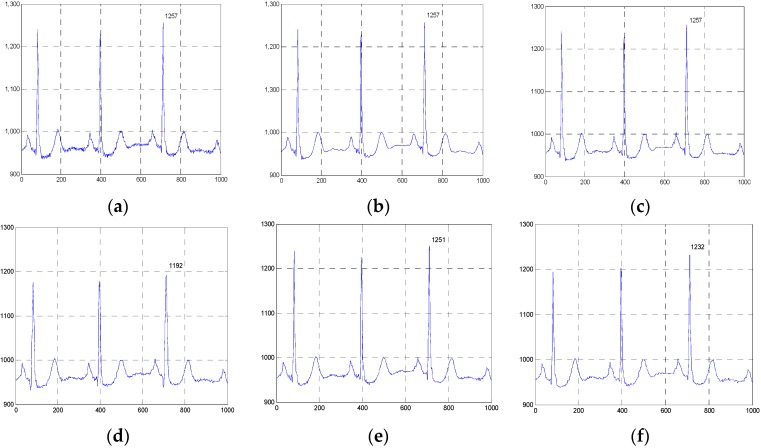
Comparison of the denoised results for all evaluated methods operating on the 100m.dat in the first group of clinical ECG signals. (**a**) The clinical ECG signal, (**b**) the ESMD-NLM method, (**c**) the NLM method, (**d**) the EEMD method, (**e**) the VMD method, (**f**) the MED filter.

**Figure 11:**
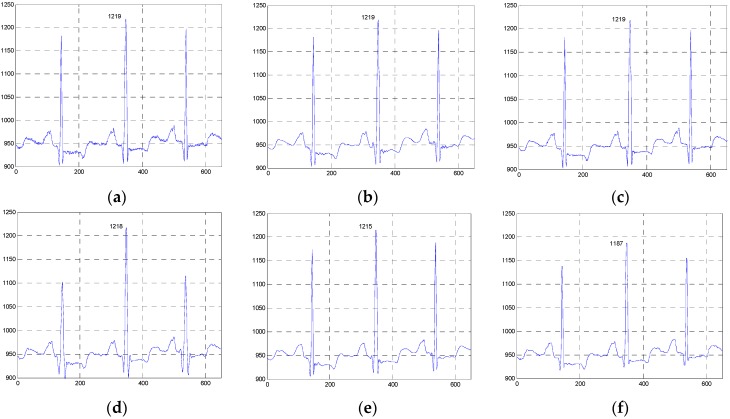
Comparison of the denoised results for the ESMD-NLM, NLM, EEMD, VMD and MED methods operating on the sel100m.dat in the second group of clinical ECG signals. (**a**) The clinical ECG signal, (**b**) the ESMD-NLM method, (**c**) the NLM method, (**d**) the EEMD method, (**e**) the VMD method, (**f**) the MED filter.

**Figure 12:**
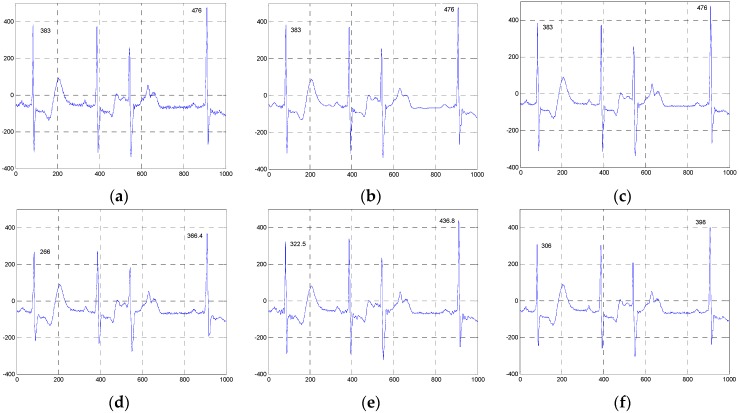
Comparison of the denoised results for the ESMD-NLM, NLM, EEMD, VMD and MED methods operating on the 13420_12m.dat in the third group of clinical ECG signals. (**a**) The clinical ECG signal, (**b**) the ESMD-NLM method, (**c**) the NLM method, (**d**) the EEMD method, (**e**) the VMD method, (**f**) the MED filter.

**Figure 13:**
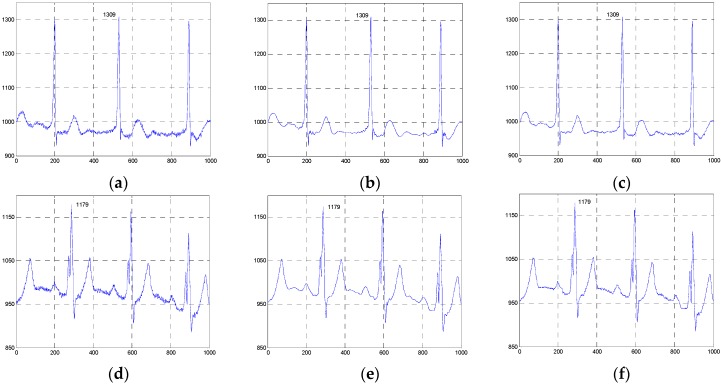
Comparison of the denoised results for the ESMD-NLM and NLM methods operating on the 111m.dat and 221m.dat in the first group of clinical ECG signals. (**a**) The clinical ECG signal (111m.dat), (**b**) the ESMD-NLM method (111m.dat), (**c**) the NLM method (111m.dat), (**d**) the clinical ECG signal (221m.dat), (**e**) the ESMD-NLM method (221m.dat), (**f**) the NLM method (221m.dat).

**Figure 14:**
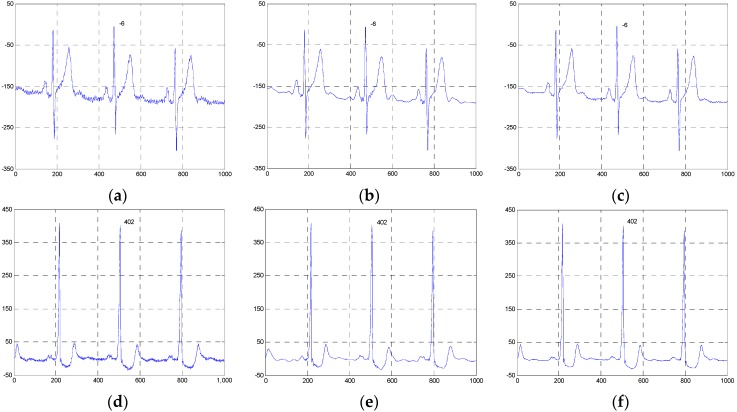
Comparison of the denoised results for the ESMD-NLM and NLM methods operating on the sel117m.dat and sel114m.dat in the second group of clinical ECG signals. (**a**) The clinical ECG signal (sel117m.dat), (**b**) the ESMD-NLM method (sel117m.dat), (**c**) the NLM method (sel117m.dat), (**d**) the clinical ECG signal (sel114m.dat), (**e**) the ESMD-NLM method (sel114m.dat), (**f**) the NLM method (sel114m.dat).

**Figure 15:**
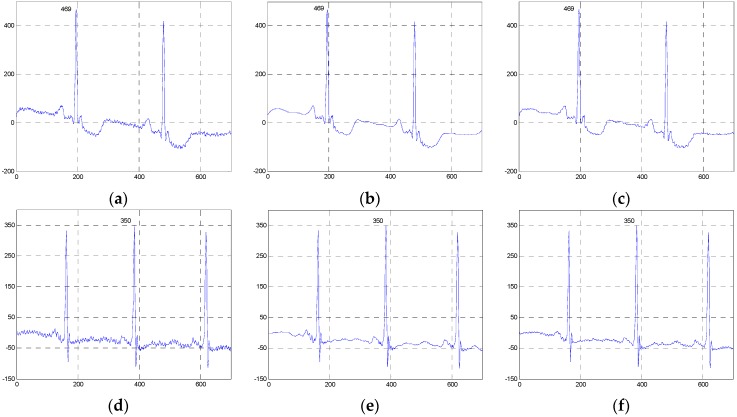
Comparison of the denoised results for the ESMD-NLM and NLM methods operating on the 13649_04m.dat and 12713_04m.dat in the third group of clinical ECG signals. (**a**) The clinical ECG signal (13649_04m.dat); (**b**) the ESMD-NLM method (13649_04m.dat); (**c**) the NLM method (13649_04m.dat); (**d**) the clinical ECG signal (12713_04m.dat); (**e**) the ESMD-NLM method (12713_04m.dat); (**f**) the NLM method (12713_04m.dat).

Figure 10, Figure 11 and Figure 12 show the signals from 100m.dat, sel100m.dat and 13420_12m.dat and the denoised results for all evaluated methods, respectively. Figure 13, Figure 14 and Figure 15 show the signals from 111m.dat and 221m.dat, sel117m.dat and sel114m.dat, 13649_04m.dat and 12713_04m.dat as well as the denoised results for the proposed method and the NLM method, respectively. From Figure 10, Figure 11 and Figure 12, we can see that the ESMD-NLM method provides amplitude values of the denoised signals much closer to those of the clinical ECG signals than the EEMD, VMD and MED methods, which demonstrates that the proposed method performs better in preserving signals’ amplitudes than the latter. Meanwhile, it can be seen from Figure 10, Figure 11, Figure 12, Figure 13, Figure 14 and Figure 15 that the ESMD-NLM method can suppress noise in the smooth segments in the ECG signals more effectively than the other four methods. Indeed, the visual comparisons demonstrate the practicality and superiority of our method in denoising the different clinical ECG signals in terms of noise removal and detail preservation. 

To evaluate the performance of these compared methods operating on the real ECG signals, the MOS tests are also implemented. Because the original noise-free versions of these real ECG signals are unknown, only the clinical blind MOS tests are done by the three cardiologists without knowing the source of each tested signal. For the blind MOS tests, only the general quality score of the denoised signals is obtained for each cardiologist as described in [30] and the final quality score denoted by MOS is the average of the scores from the three cardiologists. The larger the MOS value, the higher the quality of the denoised signal is. Table 3 and Table 4 list the MOS values for all the evaluated methods operating on the three groups of clinical ECG signals and the ESMD-NLM and NLM methods operating on the six groups of clinical ones, respectively. Table 3 and Table 4 show that the ESMD-NLM method provides the highest MOS values for the nine groups of ECG signals among all evaluated methods. Therefore, the proposed ESMD-NLM method outperforms other methods in terms of the visual quality of the denoised signals, and it may facilitate more accurate diagnosis of heart disease.

### 4.3. Comparison of Computational Efficiency 

To compare the computational efficiency of all evaluated methods operating on the 100m.dat, sel100m.dat and 13420_12m.dat, Table 5 lists the computational time for these methods. Here, all the methods are implemented using MATLAB R2014a (MathWorks, Natick, Massachusetts, MA, USA) on a personal computer with 2.30 GHz CPU and 4.00 GB RAM. It is shown in Table 5 that the proposed ESMD-NLM method has higher computational efficiency than the EEMD method, but lower computational efficiency than the NLM and VMD methods. The reason lies in the fact that in the proposed method, much computational time is involved in the ESMD based ECG signal decomposition and the NLM denoising for different IMFs. Indeed, the parallel computing strategy can be adopted to greatly improve the computational efficiency of our method.

## 5. Conclusions

In this paper, we have proposed a novel ECG signal denoising method by combining ESMD with NLM. In the proposed ESMD-NLM method, ECG signals are decomposed into many IMFs with different frequencies, and then the IMFs are denoised by nonlocal means using different decay parameters based on the QRS detection results. Quantitative comparisons based on the simulated ECG signals demonstrate that the ESMD-NLM method performs better than the median filter, the wavelet based method, the EEMD method and the NLM method by providing lower PRD, RMSE and MOS error values as well as higher SNR values. Visual comparisons based on the simulated and clinical ECG signals indicate that the proposed method gains an advantage over the compared methods in terms of noise suppression and detail preservation. Future work will be focused on the extension of the ESMD-NLM method to denoising other physiological signals such as electrooculogram (EOG), electroencephalograph (EEG) and electromyogram (EMG) signals.

## Figures and Tables

**Table 1:** The mean MOS_error_ values for the two groups of simulated ECG signals filtered by all evaluated methods.

The First Group					The Second Group				
ESMD-NLM	NLM	VMD	EEMD	MED	ESMD-NLM	NLM	VMD	EEMD	MED
10	15	20	25	30	10	20	25	25	30

**Table 2:** The PRD, SNR, RMSE and MOS_error_ values for the ESMD-NLM, NLM, VMD and EEMD methods operating on the simulated ECG signal corrupted with muscle artifact noise.

Metrics	ESMD-NLM	NLM	VMD	EEMD
PRD	13.405	14.734	17.689	18.317
SNR	17.455	16.634	15.046	14.743
RMSE	5.491	6.036	7.247	7.504
MOS_error_	20	30	55	60

**Table 3:** The MOS values for the ESMD-NLM, NLM, VMD and EEMD methods operating on the three groups of clinical ECG signals.

Data	Methods				
	ESMD-NLM	NLM	EEMD	VMD	MED
100m	4.33	3.33	2.33	2.67	2.33
Sel100m	5	4	2.67	3	2.33
13420_12m	4.67	3.33	2	2.67	2

**Table 4:** The MOS values for the ESMD-NLM and NLM methods operating on the six groups of clinical ECG signals.

Methods	Data					
	111m	221m	Sel117m	Sel114m	13649_04m	12713_04m
ESMD-NLM	5	4.67	5	5	4.33	5
NLM	3.33	3.33	3.67	4.67	3.33	3

**Table 5:** The implementation time (s) for the ESMD-NLM, NLM, VMD and EEMD methods operating on the three groups of clinical ECG signals.

The Clinical ECG Signals	ESMD-NLM	NLM	VMD	EEMD
The first group (100m.dat)	65.586	11.17	21.405	407.167
The second group (sel100m.dat)	56.31	10.334	15.361	1107.875
The third group (13420_12m.dat)	54.346	11.591	17.36	764.325

## Abbreviations

The following abbreviations are used in this manuscript:

ECG: electrocardiogram
NLM: nonlocal means
EMD: empirical mode decomposition
EEMD: ensemble empirical mode decomposition
ESMD: extreme-point symmetric mode decomposition
VMD: variational mode decomposition
IMFs: intrinsic mode functions
AGM: adaptive global mean
MA: muscle artifact
EM: electrode movements
WAV: wavelet transform
MED: median filter
PRD: percent root mean square difference
SNR: signal-to-noise ratio
PSNR: peak signal-to-noise ratio
RMSE: root mean square error
MOS: mean opinion score
